# Context matters: Causality and global epidemiology of proton pump inhibitor safety

**DOI:** 10.1371/journal.pmed.1004862

**Published:** 2026-01-07

**Authors:** Shinji Okabayashi, Gilaad G. Kaplan

**Affiliations:** Division of Gastroenterology and Hepatology, Department of Medicine, University of Calgary, Calgary, Alberta, Canada

## Abstract

In this Perspective, Gilaad Kaplan and Shinji Okabayashi highlight a recent study in PLOS Medicine that presents new evidence assessing whether links between prolonged proton pump inhibitor (PPI) use and upper gastrointestinal cancer are causal.

## Teaser

Proton pump inhibitors revolutionized acid-related disorder therapy, yet widespread overuse has raised concerns about long-term safety. A recent study in *PLOS Medicine* presents new evidence assessing whether links between their prolonged use and upper gastrointestinal cancer are causal.

Since their introduction into clinical practice in the 1980s, proton pump inhibitors (PPIs) have transformed the management of acid-related disorders [1]. Their potent suppression of gastric acid secretion has made them indispensable for the treatment of gastroesophageal reflux disease and peptic ulcer disease, prevention of nonsteroidal anti-inflammatory drug–induced ulcers, and as a component of eradication therapies for *Helicobacter pylori*, the most common chronic bacterial stomach infection. The widespread use of PPIs has dramatically improved patients’ quality of life and reduced serious complications such as bleeding ulcers. Yet, this very success has led to a paradox: the habitual, long-term use of PPIs far beyond clearly defined clinical indications. For years, growing evidence has highlighted concerns over the widespread overuse of PPIs [2] and their potential adverse effects [3,4], such as kidney disease, bone fractures, infection, and upper gastrointestinal (GI) cancer, which have resulted in enduring controversies [4,5].

In this context, a recent pharmacoepidemiological study in *PLOS Medicine* [6] provides timely and rigorous evidence that revisits this long-standing debate. The authors sought to overcome the inherent limitations of observational research by employing a rigorous study design and multiple complementary analytical approaches to reassess the association between long-term PPI use and upper GI cancer. Research into the effect of long-term PPI use on upper GI cancer has been hindered primarily due to confounding by indication and time-related biases, particularly reverse causation. Confounding by indication refers to a situation in which patients receiving PPIs for GI symptoms often have preexisting GI conditions, including *H. pylori* infection, that predispose them to the subsequent development of upper GI cancer. As a result, the observed association may be distorted, making it seem as though PPI use itself contributes to carcinogenesis. Reverse causation, or protopathic bias, arises when the initiation of PPI use occurs in response to an early symptom of the undiagnosed upper GI cancer, making it seem as though subsequent cancer detection is a consequence of drug exposure.

The authors addressed these methodological challenges by utilizing a large-scale national database in Israel, identifying 875 cases with upper GI cancer and matching them to 8,750 controls. They assessed the relationship between long-term PPI use and upper GI cancer development by using a model adjusted for the underlying indications for PPI prescription, in addition to stratifying PPI exposure according to different time windows prior to cancer diagnosis. The results showed that the risk of upper GI cancer was markedly elevated within a year preceding cancer diagnosis, especially for exposure occurring 0–0.1 years (adjusted odds ratio, ranged 3.61–8.28) and 0.1–0.5 years (adjusted odds ratio, ranged 2.73–5.72) before diagnosis, corresponding to the period when early symptoms of undiagnosed cancer are most likely to appear. The risk of upper GI cancer diminished progressively with increasing time since last use, and no excess risk was observed for PPI use occurring more than 1 year prior to diagnosis. These findings imply that the observed association between long-term PPI use and upper GI cancer likely reflects confounding by indication and reverse causation, representing a clear methodological progress over previous studies. This is consistent with recent meta-analyses that addressed this issue with more rigorous designs and analytical strategies, including meta-analyses that synthesized only studies with comprehensive confounding adjustment and those that conducted sensitivity analyses excluding low-quality studies, which frequently reported attenuated or null associations, suggesting that some alarming findings may reflect methodological artifacts rather than true drug toxicity [4,7].

However, like all observational studies, the current research cannot completely rule out the limitations inherent in this design, such as residual confounding, biases other than those mentioned above, and misclassification of exposure or disease, and the findings should be interpreted cautiously in terms of causality. It should also be noted that randomized controlled trials, while the gold standard for causal inference, are rarely feasible for this issue because of ethical and logistical constraints such as limited sample sizes, short follow-up periods, and restricted study populations, making it difficult to establish a definitive causal relationship. Furthermore, the specific findings regarding the association between PPIs and upper GI cancer must be interpreted within a broader global and biological context. The mechanisms by which PPIs might influence upper GI cancer—through acid suppression that induces hypergastrinemia and perturbs the gastric microbiota, especially in individuals infected with *H. pylori* or after its eradication [8,9]—are likely to interact with environmental and host factors that differ across regions. Therefore, the impact of PPIs on upper GI cancer is likely to vary substantially across regions and populations, depending on their underlying risk profiles. In Asia, where the burden of upper GI cancer is among the world’s highest [10], the situation of *H. pylori* infection, genetic susceptibility, and lifestyle habits such as smoking creates a fundamentally different background risk compared with Western populations. These biological and environmental heterogeneity may function as an effect modifier in the association between PPI use and upper GI cancer, shaping both the emergence and the strength of the observed relationship across regions. Furthermore, when evaluating the magnitude of effects across regions, it is essential to account for the underlying baseline risk of the disease within each population [11]. In countries such as those in East Asia, where the incidence of upper GI cancer is substantially higher than populations in other regions [10], even a similar relative risk can translate into a much greater absolute risk. In other words, the number of individuals who need to be exposed to cause one additional adverse event (i.e., the number needed to treat for an additional harmful outcome) decreases in populations with a higher baseline risk, resulting in a more substantial public health burden. A “one-size-fits-all” interpretation of PPI safety data may obscure these critical epidemiological nuances.

**Fig 1:**
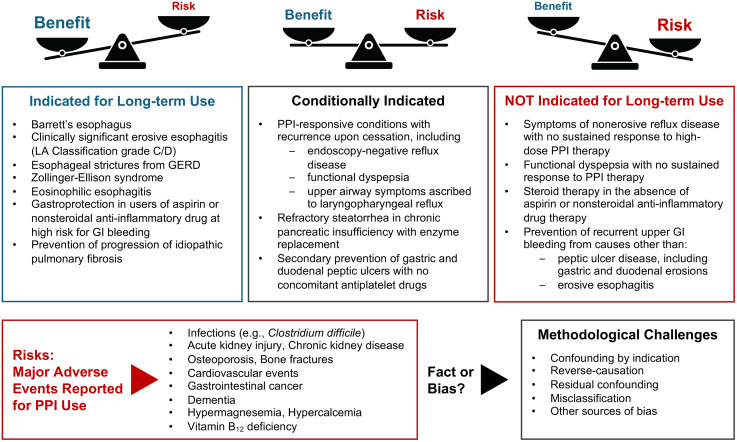
Indications for long-term proton pump inhibitor use considering the risk of potential adverse events. Indications for long-term proton pump inhibitor (PPI) use are provided based on the framework proposed by the American Gastroenterological Association Clinical Practice Update [12]. Potential adverse events related to long-term PPI use have been reported for several conditions; however, most are not supported by evidence strong enough to establish a causal relationship because of methodological challenges.

The evidence on long-term adverse effects of PPIs remains uncertain, underscoring the need for continued high-quality studies that rigorously address confounding, time-related biases, and other methodological limitations. At the same time, there is no doubt that PPIs remain highly effective and indispensable agents for the management of acid-related disorders. The best current practice is to ensure the appropriate use of PPIs by carefully balancing their potential long-term risks with the expected therapeutic benefits (Fig 1), while taking into account individual patient profiles and regional epidemiologic contexts. Given the widespread overuse of PPIs, clinicians should periodically reassess the need for continued therapy and screen for potential adverse effects, while considering de-prescribing [12] or alternative strategies such as switching to lower-risk medications and promoting healthier lifestyle habits. Such a comprehensive and cautious approach is key to maximizing the therapeutic benefits of PPIs while minimizing the potential risk of adverse events.
